# Tomatoes

**Published:** 1859-09

**Authors:** 


### TOMATOES.

WITHIN our memory this vegetable was considered a mere ornament for the mantelpiece and a pretty plaything for the children. We grew to manhood and never saw it on our father's table—nor onions—yet the onion is one of the most nutritious vegetables which springs from the earth, while the tomato has become an almost universal edible; it is equally palatable whether raw, cooked, or preserved, and has the reputation of being the most harmless and healthful of all the articles sold by the green-grocer, from August until frost.

The *Working Farmer* says of the tomato plant, that it "bears eighty per cent. of its fruit within eighteen inches of the ground, while more than half of the plant is above that part. When the branches are cut they do not bleed, and they may therefore be shortened in immediately above the large or early setting fruit.—The removal of the small fruit on the ends of the branches is no loss, for the lower fruit will swell to an unusual size by the trimming, and both a greater weight and measure of fruit will be the consequence, beside obtaining a larger portion five to fifteen days earlier. The trimming should be so done as to leave a few leaves beyond the fruit, to insure perfect ripening. When tomatoes are first brought to market, they bring frequently four dollars per basket, and in ten days fall in price to fifty cents. The importance of early maturing is too evident to need comment. The burying of the removed portions immediately around the plant is a good practice, both by insuring full disturbance of the soil, and by the presenting a fertillizer progressed precisely to the point of fruit-making. The portions buried decay rapidly, and are readily assimilated.

